# Unveiling of Pyrimidindinones as Potential Anti-Norovirus Agents—A Pharmacoinformatic-Based Approach

**DOI:** 10.3390/molecules27020380

**Published:** 2022-01-07

**Authors:** Oluwakemi Ebenezer, Nkululeko Damoyi, Maryam A. Jordaan, Michael Shapi

**Affiliations:** Department of Chemistry, Faculty of Natural Science, Mangosuthu University of Technology, 511 Mangosuthu Highway, Durban 4000, South Africa; nedamoyi@gmail.com (N.D.); maryamamrajordaan@gmail.com (M.A.J.); mshapi@mut.ac.za (M.S.)

**Keywords:** human norovirus, virtual screening, multiple docking, molecular dynamic

## Abstract

The RNA-dependent RNA polymerase (RdRp) receptor is an attractive target for treating human norovirus (HNV). A computer-aided approach like e-pharmacophore, molecular docking, and single point energy calculations were performed on the compounds retrieved from the Development Therapeutics Program (DTP) AIDS Antiviral Screen Database to identify the antiviral agent that could target the HNV RdRp receptor. Induced-fit docking (IFD) results showed that compounds ZINC1617939, ZINC1642549, ZINC6425208, ZINC5887658 and ZINC32068149 bind with the residues in the active site-B of HNV RdRp receptor via hydrogen bonds, salt bridge, and electrostatic interactions. During the molecular dynamic simulations, compounds ZINC6425208, ZINC5887658 and ZINC32068149 displayed an unbalanced backbone conformation with HNV RdRp protein, while ZINC1617939 and ZINC1642549 maintained stability with the protein backbone when interacting with the residues. Hence, the two new concluding compounds discovered by the computational approach can be used as a chemotype to design promising antiviral agents aimed at HNV RdRp.

## 1. Introduction

At times, norovirus can be referred to as a winter vomiting bug or food poisoning and is the most common cause of gastroenteritis contagion in mammalian hosts. People infected with norovirus develop symptoms from between 12 to 48 h. The symptoms include diarrhea leading to dehydration, vomiting, nausea, and stomach pain. These viruses have their place in the *Caliciviridae* family with non-enveloped particles, 27–40 nm in diameter. Most norovirus genomes are comprised of three open reading frames (ORFs) apart from murine noroviruses, which contain a fourth ORF [1]. ORF1 encodes the polyproteins that are cleaved into six non-structural proteins (p48 (NS1/2), NTPase (NS3), p22 (NS4), VPg (NS5), along with the virus-encoded 3C-like cysteine protease (3CLpro) and a viral RNA-dependent RNA polymerase (RdRp) [2]. Among these nonstructural proteins, RdRp has been reported to play a crucial role in genome replication, as well as in the synthesis and amplification of additional subgenomic RNA [3]. ORF2 comprises the main capsid protein (VP1), which has a shell (S) and protruding (P) domains. The S domain environs the viral RNA, and the P domain, which consists of the P2 domain, is connected to the S domain through a flexible hinge [1,4,5]. Highly diverse norovirus genus are classified into seven distinct genogroups. The wide-reaching outburst of human norovirus (HNV) infection has been largely caused by genogroup II and genotype 4 (GII.4) strains. According to the Centers for Disease Control and Prevention, norovirus brings about ~685 million cases globally, and ~200 million incidents are observed among children under the age of five, leading up to ~50,000 child deaths annually and frequently in developing countries. Moreover, norovirus causes about 19 to 21 million episodes of illness, and ~900 deaths, mostly among adults above the age of 65, ~109 thousand hospitalizations, and ~465 thousand emergency office visits, mainly for young children, in the United States annually. The main routes of NoV transmission include ingestion of contaminated water or food and contact with contaminated surfaces or infected persons. Some evidence also suggests that norovirus may be transmitted through the air [6,7]. Moreover, the evaluation modeling of a norovirus vaccine’s cost-value and economics is unsurprisingly satisfactory due to the illness burden [8]. The prolonged absence of a cell culture system and reduced animal models have made anti-norovirus drug design and vaccines impeded; however, several broad antiviral agents have effectively acted positively against norovirus. For example, ribavirin, a nucleoside analog, has helped treat a respiratory syncytial virus, Lassa virus, hepatitis C virus (combined with pegylated interferon) and have prevented imitation of norovirus in the Norwalk replicon model [9,10]. Similarly, 2′-C-methylcytidine inhibited viral replication with an EC50 value of 2 µM and constrained viral replication at non-toxic concentrations in in vitro murine norovirus, a surrogate for human norovirus [10]. Kolawole et al. reported the effectiveness of 2′-C-methylcytidine in HNV infection using cell culture and a mouse model [11]. Favipiravir is an antiviral molecule used for the medication of influenza virus contagions. Besides, the molecule is active against distinct RNA viruses, such as bunyaviruses, arenaviruses, and flaviviruses [12,13,14], and it also inhibits in vitro murine norovirus replication [15]. Arias et al. evaluate the effectiveness of favipiravir in mice infected with norovirus. The output indicated an increase in the number of mutations that occurred when the viral RNA replicated and could reduce the infectious virus’s extent isolated from feces in MNV-infected mice to imperceptible amounts. The absence of toxicity was observed after eight weeks of therapy [16]. Nevertheless, these compounds’ mode of action ensued in off-target side effects and we were unable to get to clinical trials for norovirus therapy [17]. CMX521, a derivative of sangivamycin, is a nucleoside analog of ribofuranose [2]. This molecule is another nucleoside analog of ribofuranose that potently suppresses murine norovirus (MNV) in mice and was the first nucleoside analog to be moved to clinical trials (phase 1) for the prevention and treatment of human norovirus [2,18].

Additionally, nonnucleoside compounds, such as suramin, NF203, and PPNDS, inhibit human norovirus, including suramin-related compounds. Toxicity hitches have previously taken back these compounds’ development; variations in the suramin structure lessened the toxicity but maintained the ability to inhibit both human and murine norovirus RdRp effectively [19]. Nitazoxanide is a nonnucleoside compound that showed an excellent anti-norovirus inhibitory effect in clinical trials, but the specific mechanism against norovirus infection has remained unidentified. Based on the threat of norovirus to humankind and the lack of drugs to combat this virus, there is an urgent need to search and develop potent, specific, and noncytotoxic inhibitors of HNV RdRp with enhancing therapeutic clinical value. However, developing new drugs is scientifically challenging, time-exhausting, and expensive. However, the use of computer-aided methodologies for drug design of new hit compounds is fast and cost-effective. Hence, high throughput virtual screening (HTVS) of an Antiviral Screen Database designed by the National Cancer Institute (NCI) branch of National Institutes of Health (NIH), collectively with e-pharmacophore screening, single-point energy calculations, and molecular dynamics studies, were used to unveil potential anti-norovirus agents.

## 2. Material and Methods

### 2.1. Softwares Used in This Study

Molecular modeling software from Schrödinger’s Drug Discovery Suite 2020-4 (Schrödinger, Inc., LLC, New York, NY, USA) and Discovery Studio Visualizer software created by Accelrys (Dassault Systemes, BIOVIA Corp., San Diego, CA, USA) were used.

### 2.2. Chemical Library Dataset

The compounds used in this study were downloaded from Antiviral Screen Database established by NCI [20]. Importantly, there is no evidence of stereochemical information and geometry optimization performed on the screened compounds in the database [20]. A total of 42,390 were retrieved for further analysis.

### 2.3. Ligand Preparation

The retrieved ligands were laid open to preparation using the Ligprep module of the Schrodinger Suite. The 2D compounds were converted to 3D structures and optimized using the OPLS (optimized potentials for liquid simulations) all-atom 2005 force field, generating the lowest possible energy conformation. Tautomers with substantial populations for each input structure were generated, and ionization on a cellular pH value (7.0 ± 2.0), i.e., setting the panel to include the original ionization state to the generated states using the Epik tool [21,22,23].

### 2.4. Receptor Preparation and Grid Generation

HNV RdRp protein’s crystal structure with its native ligand PPND was retrieved from the protein data bank (PDB; 4LQ3) with a resolution of 2.5 Å and imported into the protein preparation wizard. RdRp is the key enzyme in the viral biological cycle of all RNA viruses, irrespective of the polarity of the viral RNA genome [24]. In addition, there is a lack of RdRp in mammalian cells, which makes the enzyme acts as an appropriate target for inhibition in the perspective of antiviral prophylaxis [19]. First, the protein was preprocessed by adding bond orders, adding hydrogen atoms to the structure, and modifying metal ionization states to certify proper formal charge and force field treatment. Next, crystallographic water molecules beyond 5 Å were removed, creating disulfide bonds, cap protein termini with ACE (N-acetyl) and NMA (N-methyl amide) groups; heteroatoms were done using the Epik tool. After reviewing and modifying the protein and the co-crystal ligands, the protein’s hydrogen bonds were optimized and minimized. Finally, the receptor grid file generation was done using a Glide module route (Grid-based Ligand Docking with Energetic) [25].

### 2.5. Virtual Screening and E-Pharmacophore Generation

A virtual screening workflow panel in the Schrodinger suite was used for these steps. The ligands were prefiltered by the Lipinski rule and the reactive functional groups option. The output ligands were passed for e-pharmacophore screening. In this step, the native ligand of HNV RdRp was docked into the receptor’s binding site using glide with the extra-precision mode (XP). Therefore, the protein–ligand complex was used as input for generating pharmacophore sites in the Phase module embedded in the Schrodinger suite [26]. The generated hypothesis was used to screen the prepared ligands. The docking section includes three stages, and the first stage performs HTVS docking. In the HTVS approach, the bad hits are removed with ease, and the remaining compounds are recalled and moved to the next docking stage, which is SP (standard precision) docking. The stage survivors are retained and moved to the third stage, which performs XP (extra precision) docking and removes false positives that pass through the glide SP [27].

### 2.6. Induced Fit Docking

The virtual screening final compounds were passed through induced fit docking (IFD) in the Schrodinger software suite to improve the docking accuracy and find a better docking pose [28]. The IFD protocol used in this study was carried out in three consecutive steps. The preliminary step is the ligands’ docking into the receptor’s rigid active site using Van der Waals radii scaling of 0.5 for protein and ligand. Standard precision was used during the initial docking and extra precision for the final redocking; thus, by default, a maximum of 20 poses was to be carried forward for each ligand after initial docking. Besides, the ligands posing with residues of at least one atom within 5 Å were subject to a conformational search and minimization. In contrast, the remaining residues outside the 5 Å were fixed; hereafter, the plasticity of proteins was considered. The refined complexes were ranked by prime energy, and the receptor structures within 30 kcal/mol of the minimum energy structure were passed through for a final glide docking and scoring round. An IFD score for the protein–ligand contacts energy and the system’s binding energy was predicted and used to rank each output pose. The more negative the glide score, the more favorable the ligands’ binding to the active site residues.

### 2.7. Quantum Mechanical/Molecular Mechanical (QM-MM) Using Qsite

The hit compounds were evaluated further to study the details of their electronic properties. The electronic properties, such as molecular electrostatic surface potential (MESP), highest occupied molecular orbitals (HOMO), and lowest unoccupied molecular orbitals (LUMO), were calculated in this study. The geometry optimization of the HNV RdRp-ligands docking poses was done using the QM-MM approach without any constraints in the gas-phase in Qsite implemented in the Schrodinger software suite, and the non-interacting proteins were in the MM region [29,30]. The frozen-orbital method was applied to atoms in both regions. The ligands were treated with quantum mechanics (QM), and the receptor was treated with molecular mechanics (MM) using hydrogen cap electrostatics to separate the two regions. Electrostatic embedding was used. Each of the estimates in the Qsite was accomplished using DFT with B3LYP (Berke’s three-parameter exchange potential and the Lee-Yang-Parr correlation functional) and using the LACVP* basis set [31].

### 2.8. Molecular Dynamics Simulations

The hit compounds were subjected to a molecular dynamic simulation to investigate the stability binding of the ligands in the active pocket of HNV RdRp using Desmond software. The molecular dynamic simulation protocols are as follows. The system builder preference was used to fit the simple point charge (SPC); the water model was contained in the protein–ligand complex in an orthorhombic periodic boundary of the box volume [32]. Meanwhile, chlorine ion was included for the neutralization of the system, which varies as per the system’s total charge, after enclosure with a salt concentration of 0.15 M. Molecular simulations were accomplished with a periodic boundary condition in the number of atoms, pressure, and temperature (NPT) ensemble. The OPLS forcefield was applied for calculations. All systems were subjected to Desmond’s default eight-stage relaxation protocol before the start of the production run [33]. The temperature was at 300 K, one atmospheric pressure, and all the complexes, were capitulated to a run of 100 ns.

## 3. Results and Discussion

The crystal structure of HNV RdRp consists of a site-A and site-B. Meanwhile, the B-site is a vastly conserved binding site within the thumb region and can be exploited to scheme and develop novel antiviral agents against human norovirus [19]. The PPNDS forms interactions with residue Arg392, Gln414, Ser410, Val504, Leu406, Glu506, and Asp507. The binding pocket of HNV RdRp with its co-crystallized ligand was analyzed by SiteMap [34] (Figure S1), which shows a potential hydrogen bond donor, a hydrogen bond acceptor, and a hydrophobic and hydrophilic surface. The predicted hydrophilic mapped regions can be classified into the hydrogen-bond donor, the hydrogen-bond acceptor, and metal-binding regions, and the protein’s surface is contoured.

**Figure 1:**
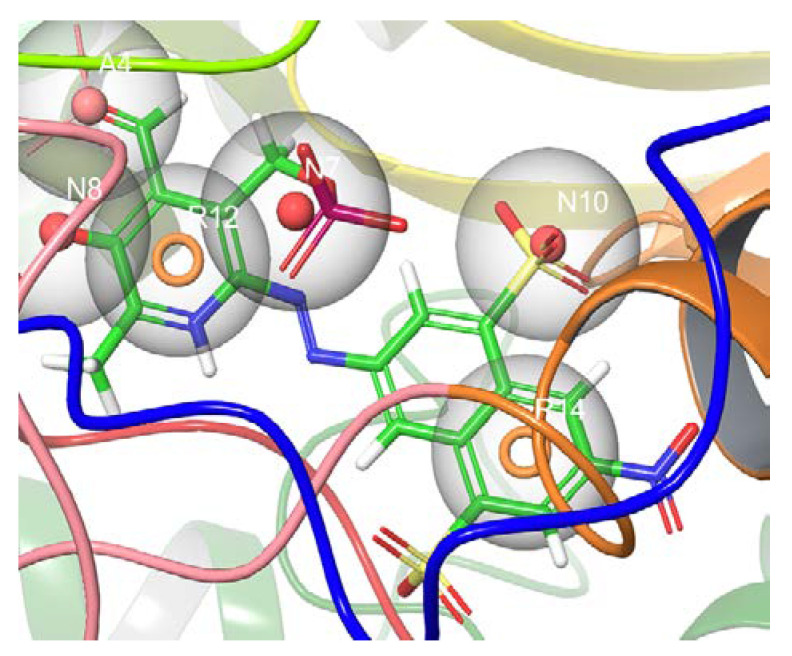
The predicted model based on PPNDS binding to the human norovirus RdRp 4LQ3 crystal structure and excluded volume is shown in grey. The model consists of three anionic groups (N7, N8, and N10), two aromatic features (R12 and R14), and one hydrogen bond acceptor (A4).

The co-crystallized ligand was docked into the active site’s HNV RdRp receptor using the Glide XP method to generate a pose viewer file. It was found that the glide XP method reproduced the native ligand conformation of the docked ligand. The protein–ligand complex was used further as an input for the e-pharmacophore to create a hypothesis (Figure 1). The Glide XP scoring terms were used to uncover which features mostly influence the binding. The docking score, glide emodel, and glide energy values of the docked complex correspond to −8.653, −77.213, and −63.332.

Furthermore, the generated hypothesis was used to filter small molecule databases; namely, AIDS Antiviral Screen Database, consisting of 42,390 chemical structures checked for evidence of in vitro anti-HIV activity. Before the e-pharmacophore screening, the compounds were subjected to ligand preparation using the Ligprep module of the Schrodinger Suite. Thus, ionization states and tautomeric forms of the structures at pH 7.0 ± 2.0 were generated. A total of 61,365 compounds were retrieved, and 2477 compounds were dropped structures in this step. Lipinski’s five (RO5) rule was further used to eliminate less appropriate compounds. The rule states that a drug-like molecule has less than five hydrogen bond donors and not more than ten hydrogen bond acceptors. Molecular weights of below 500 g/mol and the calculated LogP (clogP) should not be more than 5 (or QlogP < 5), as structures that fulfill this optimum prerequisite are considered drug-like, and structures that do not satisfy this rule are removed [35]. In addition, the filtering option was set to filter out ligands that have reactive functional groups. Out of 61,365 compounds, 30,856 compounds that fulfill the optimum prerequisite for drug-like and the reactive functional groups are well-thought-out as active compounds for HNV RdRp. A total of 30,856 compounds were extracted and used for further analysis.

Virtual screening workflow in the Schrodinger Suite was employed for the structure-based virtual screening using three types of docking methods (HTVS, Glide SP, and Glide XP). The resulting compounds were first passed through HTVS. The docking section used the HTVS mode to eliminate the compounds with bad hits faster and return 15% of the compounds (4714) based on the highest glide score. Furthermore, e-pharmacophore-based screening was utilized to screen the compounds and is based on the phase fitness score; 468 compounds are returned as active. In addition, Glide SP was utilized and 50% of the docked ligands (235) were passed to glide XP mode for docking. The Glide XP mode applied a more complex scoring function than the HTVS and Glide SP. This step eliminates false positives compounds that pass through the Glide SP; moreover, Glide XP will penalize the ligands that are not suitable for the receptor; the protocol was set to return 50% of the docked ligands (117).

Additionally, induced-fit docking (IFD) was performed to corroborate and enhance the compounds’ interaction in the HNV RdRp receptor’s binding pocket. The most significant facet of induced-fit docking (IFD) is the generation of precise, complex structures for the ligands and the residue in the receptor’s binding pocket in a flexible approach, consequently eliminating false antagonistic ligands. The five compounds with the best glide score (IFD) were selected as the hit compounds for further studies. Pharmacokinetics can be used to determine the early fate of a compound. It gives an insight into the absorption, distribution, metabolism, excretion, and toxicity (ADMET) properties of compounds in the human body before advancing to the investigational procedures. QikProp generates physically relevant descriptors and uses them to perform ADMET predictions [36]. The best five compounds from the QikProp analysis are detailed in Table 1 below, while Table 2 displays the IFD results for the hit compounds.

### 3.1. The Binding Pose of CMX521 and PPNDS—HNV RdRp Docked Complex

**Figure 2:**
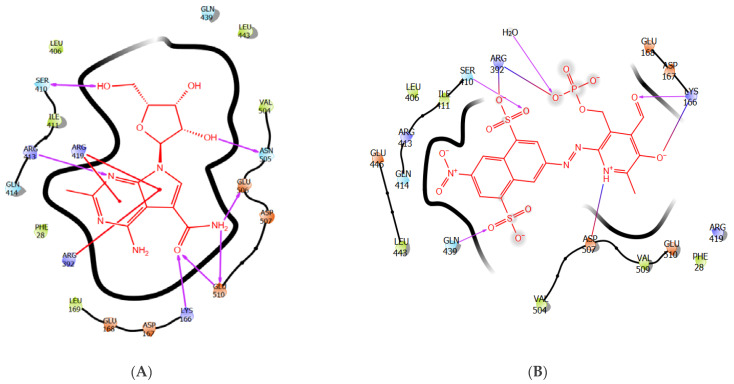
The 2-Dimensional representation of compound (**A**) CMX521 and (**B**) PPNDS in the binding pocket of HNV RdRp receptor.

Interestingly, the binding landscape of CMX521 in the active site of HNV RdRp was like the binding landscape of PPNDS, as shown in Figure 2a,b. Analysis of the binding mode of CMX521 revealed strong hydrogen bonding between the nitrogen atom of the pyrimidine ring and Arg413; meanwhile, the ring and the pyrimidine-pyrrole ring formed electrostatic interactions with Arg419 and Arg392, respectively. Furthermore, complex-stabilization was provided by H-bond interactions between the amine group of CMX521 and amino acid Glu506 and Glu510, whereas the oxygen atom of the carbonyl group interacts with Lys166 (Figure 2a). At position 1 of ribonucleoside moiety, the hydroxyl substituent established H-bonds with Asn505, whereas the hydroxyl methyl interacted with Ser510. Moreover, the amine group of PPNDS formed a salt bridge with Asp507, while H-bond interactions were observed between the oxygen atoms and side chains of Arg392 and Ser410. Besides, Lys166 was inserted between the carbonyl functional group and the oxygen atom of the pyridine ring of PPNDS to formed an H-bond interaction and salt bridge (Figure 2b). The oxygen and the nitrogen atoms of PPNDS interact more with the residues compared to the sulphur atoms.

### 3.2. The Binding Pose of ZINC1617939–HNV RdRp Docked Complex

**Figure 3:**
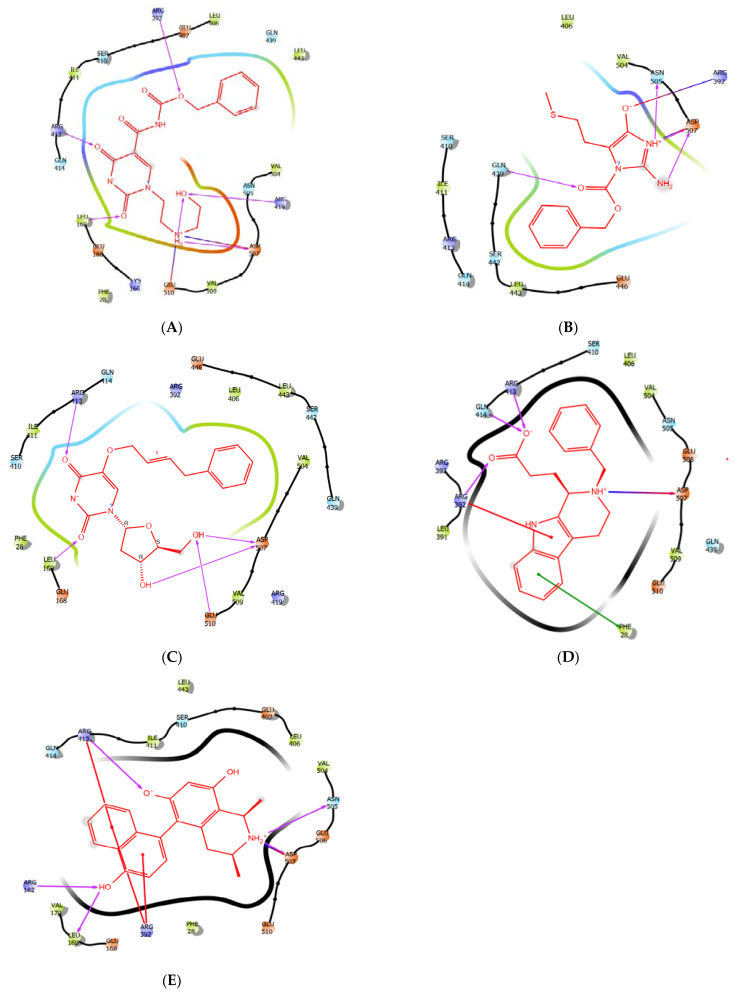
(**A**) The 2-Dimensional representation of compound ZINC1617939. (**B**) The 2-Dimensional representation compound ZINC6425208 in the binding pocket of HNV RdRp. (**C**) The 2-Dimensional representation compound ZINC1642549 in the binding pocket of HNV RdRp. (**D**) The 2-Dimensional representation compound ZINC5887658 in the binding pocket of HNV RdRp. (**E**) The 2-Dimensional representation compound ZINC32068149 in the binding pocket of HNV RdRp.

As described in Figure 3a, ZINC1617939 binds to the active site of HNV RdRp through hydrogen bonds (H-bonds), electrostatics, and hydrophobic bonds. The two-carbonyl oxygen atoms of pyrimidinedione moiety formed H-bond interactions with residues Leu169^1.73^ (amine group), Glu168^2.89^ (hydrogen atom of the amine group), and Arg413^1.73/2.96^ (methyl and amino group), respectively. Hydrophobic interactions were observed between the pyrimidinedione moiety and the methyl group of Leu169. The ionic bond resulted from the electrostatic attraction between positively charged side chains of amino acids Arg413 and the pyrimidinedione’s negative charge nitrogen atom (aromatic amine). The glide score, glide energy and glide emodel for ZINC1617939 correspond to −13.027, −81.298, and −60.302, respectively (Table 2). The interaction of ligands in the receptor active site can sometimes be a function of the binding affinity. Besides, the results from a ligand with a superior binding affinity showed that there are greater attractive forces between the ligand and its receptor. In contrast, inferior affinity ligand binding implies a reduction in the attractive force. Notably, benzyl oxycarbonyl in compound ZINC1617939 displayed three H-bond interactions accompanied by two hydrophobic interactions. The oxygen atom and carbonyl form H-bonds with the amine functional group of Arg392^2.40^ and the carboxyl oxygen atom of Asn505^2.62^; the methyl forms H-bonds with the methyl group of Ser410^2.69^. The benzyl group was inserted between residue Val504 and Leu443 and allowed hydrophobic interactions with the benzyl ring.

Interestingly, Asp507 was inter-switched between the methyl and amine group of the hydroxyethylamino to form an H-bond and a salt bridge; this interaction was observed in the binding mode of PPNDS. In contrast, the oxygen atom of hydroxyl functional group forms H-bonds with residue Glu510^2.75^ and Arg419. The amine also forms an ionic bond with the well-positioned negatively charged carboxylate ion of Glu510^2.30^.

### 3.3. The Binding POSE of ZINC6425208–HNV RdRp Docked Complex

The visual inspection of ZINC6425208–HNV RdRp (Figure 3b) showed that the benzyl ring from benzyl formate formed hydrophobic interactions with the methyl group of Val504^4.28^, Leu443^3.91^ and Ile411^5.39^; meanwhile, carbonyl oxygen forms an H-bond with the amine group of Gln439^2.02^. Undoubtedly, the anticipated binding modes of these compounds in HNV RdRp show the interaction of a secondary amine of the imidazole ring with the carboxyl oxygen atom of Asn505^2.35^ via an H-bond and electrostatic contact with Asp507. Besides, the primary amine attached to the imidazole ring H-bonded with residue Asp507^2.11^. A salt bridge was observed between the cationic ammonium from the guanidinium of Arg392 and the negatively charged oxygen atom of the hydroxyl functional group. The methyl group of the SCH_3_ forms hydrophobic interaction with residue Leu406. The interaction with the residues Ile441 and Leu406 is not permitted in the ZINC1617939-HNV RdRp binding mode, while Arg392 assumes a different interaction. The functionality group of ZINC6425208 is different from ZINC1617939; the imidazole ring’s presence decreases the binding affinity and reduces interactions with active site residues, which we think might be indispensable in enhancing the ligand binding and conferring selectivity in designing a critical experiment.

### 3.4. The Binding Pose of ZINC1642549–HNV RdRp Docked Complex

Furthermore, the interaction depiction of ZINC1642549–HNV RdRp (Figure 3c) shows that the phenyl group was well fitted into the hydrophobic pocket Leu443^4.01^, Ile411^5.37,^ and Val504^5.00,^ and the methyl group of these residues contact the phenyl ring. Remarkably, the two carbonyl oxygen atoms form H-bonds with Glu168^2.89^ (methine group), Leu169^1.72^ (amine group), and Arg413^1.75^ (amine group). The pyrimidinedione ring forms a hydrophobic interaction with residue Leu169^5.25^. Meanwhile, the nitrogen atom between the two carbonyl oxygens in the pyrimidinedione ring form ionic interactions with amine functional groups of Arg413^4.57^ and Arg392^5.10^. Furthermore, the 2,3-hydroxyl of the nucleoside H-bonded with residue Asp507^1.92^ and Glu510^2.09^. The binding landscape of CMX521 in the active pocket of the receptor was similar to the hit compounds.

### 3.5. The Binding Poses of ZINC5887658 and ZINC32068149- HNV RdRp Docked Complex

The visualization of ZINC5887658 in the binding site of HNV RdRp (Figure 3d) shows that the weak electrophile (-COOH) formed H-bonds interact with amino acid Arg392, Gln414 and Arg 413. Meanwhile, the pyrido-indole formed salt bridge with Asp507 and π-cation interaction with Arg392. Compound ZINC32068149 formed notable interactions with Arg413, Arg392, leu169, Arg182, Asp507 Asn505, Arg413 through H-bonds, π-cations, and salt bridge. (Figure 3e)

**Figure 4:**
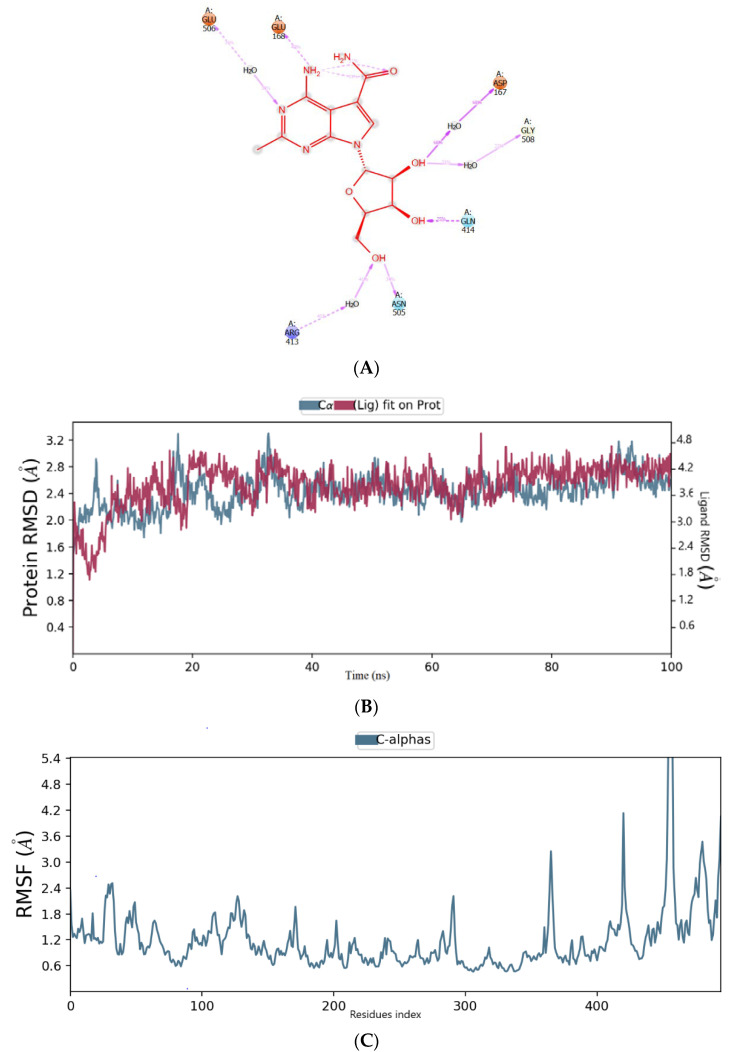
(**A**) The binding mode of CMX521 in the active pocket of HNV-RdRp receptor after MD simulations; (**B**) The graph plot root mean square deviation (RMSD) plot; (**C**) The graph plot of root mean square fluctuation (RMSF).

A molecular dynamic simulation was used to examine the interaction and conformational stability of CMX521 and the hit compounds upon binding to the active site of HNV RdRp protein. The complex was simulated for 100 ns, and the average RMSD (root mean square deviation) of HNV RdRp was found to be 2.5 Å while CMX521 (ligand) displayed an average RMSD of 2.7 Å, respectively (Figure 4a). Notably, little conformational flexibility was observed in the binding of CMX521 to HNV RdRp throughout the simulation period. Meanwhile, the root mean square fluctuation result (RMSF) of HNV RdRp protein was detailed in Figure 4b. It was observed in the plot that large fluctuations occurred at the C-terminal of the protein. Figure 4c displayed the 2D representation interactions that occur more than 30% between CMX521 and the residues of HNV RdRp protein during the simulation time. The ribonucleoside hydroxyl at positions 1 and 3 formed H-bond interactions with amino acid Ap167, Gly508, and Arg413 through water bridges. Other residues involved in the H-bond interactions include Gln414, Asn505, Glu168, and Glu506, respectively (Figure 4).

**Figure 5:**
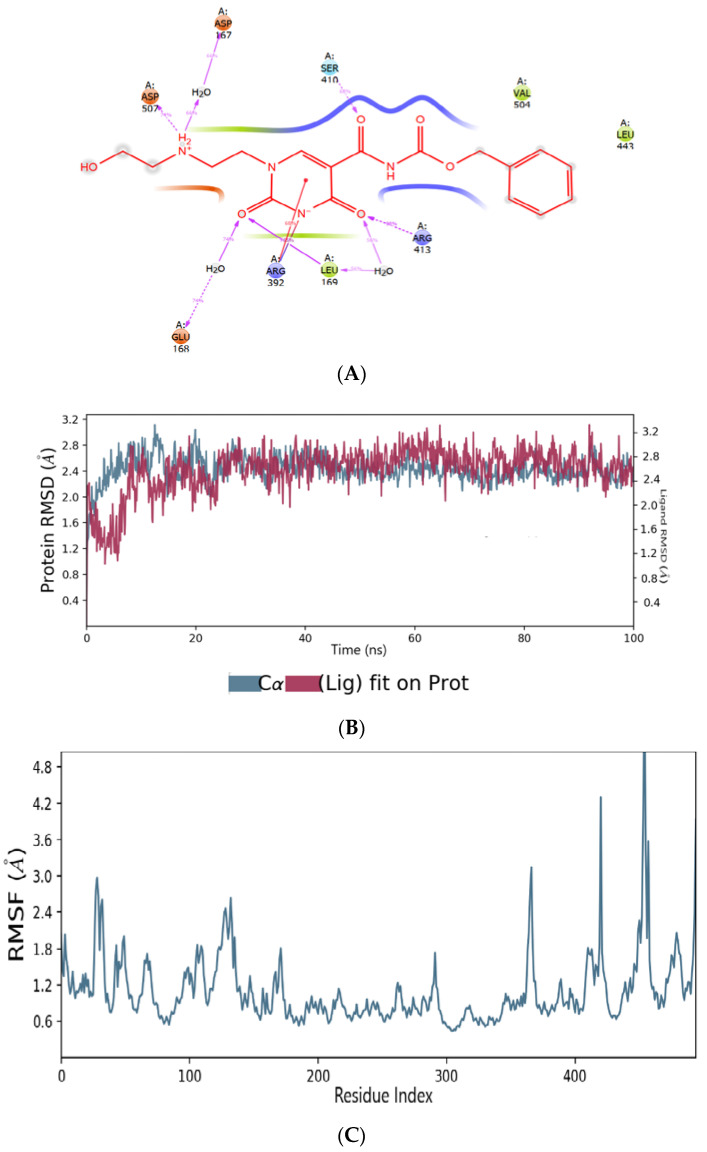

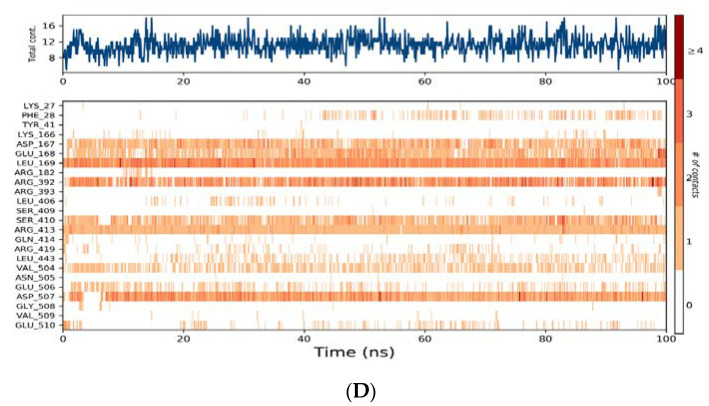
(**A**) The binding mode of ZINC1617939 in the active pocket of HNV-RdRp receptor after MD simulations; (**B**) The graph plot root mean square deviation (RMSD) plot; (**C**) The graph plot of root-mean-square fluctuation (RMSF); (**D**) A timeline representation of the interactions and contacts of ZINC1617939 in the active site of HNV RdRp receptor.

Furthermore, in the molecular dynamic studies, the simulation reached convergence at 100 nanoseconds (ns), and two out the five compounds maintained good stability during the simulation. The conformational stability of the projected structure of HNV RdRp and the poses of ZINC1617939 were evaluated from the trajectories obtained in 100 ns simulations. The analysis of the ligands’ binding site interactions with the protein residues after MD is shown in Figure 5a–c. Most of the interactions of compound ZINC1617939 and ZINC1642549 in the binding pocket of the HNV RdRp receptor are pigeon-holed by hydrophobic interactions. The benzyl oxycarbonyl in compound ZINC1617939 is still closely accommodated in the hydrophobic pocket, between helices α13 and α14 of the thumb domain HNV RdRp receptor and with the aliphatic portion of the side chain of Ser 410. However, the phenyl group lost its hydrophobic interactions while the carbonyl oxygen atom preserves its interactions with the aliphatic fragment of Ser410. Furthermore, the complex was thermodynamically stabilized by forming a salt bridge, as well as ionic interactions, between the negative charge nitrogen atom and pyrimidinedione moiety and the Arg392 residue in the palm domain of the HNV RdRp receptor. Thus, it may positively influence the anti-norovirus potency of this compound. Moreover, the two-carbonyl oxygen atoms of pyrimidinedione moiety H-bonded with Glu168 and Leu169 via a water molecule. Notably, the positively charged amine group H-bonded with Asp167 through the water bridge and sidechain of Asp507 (Figure 5a). From these observations, pyrimidindinone moiety can be explored as a chemical feature to enhance affinity and selectivity in the practical design and fusion of new inhibitors of HNV RdRp. The root mean square is commonly used to examine whether a structure is stable during the simulations or swerves from the original coordinates. The discrepancy from the original coordinates is construed to denote that the simulation is not equilibrated. RMSD deviations in the HNV RdRp receptor were noticed between 1 Å at the starting point and 2.90 Å at the 20 ns time interval. A consistent RMSD of approximately 2.5 Å was maintained during the simulation interval. Maximum numbers of deviations were noted between 12 and 20 ns. The inclusive deviations in the protein throughout simulations were steady and stayed below 3.0 Å, while the ligand displayed the same form of changes in the initial 20 ns, and no significant deviation was observed above 20ns (Figure 5b). The implication is that compound ZINC1617939 was stable and did not disturb the protein backbone stability when interacting with the residues at the HNV RdRp receptor’s active pocket. The root mean square fluctuation (RMSF) per residue was calculated. The graph was plotted alongside the residue numbers based on the trajectory time of MD simulation to investigate the protein region showing high flexibility (Figure 5c). Notably, the RMSF plot showed that residues positioned in the binding site with fluctuation lack interactions with the ligand (Figure 5c). A timeline representation of the interactions and contacts (H-bonds, hydrophobic, ionic, water bridges) is shown in Figure 5d. The top panel shows the number of specific contacts the protein makes with the ligand throughout the trajectory. The bottom panel shows which residues interact with the ligand during the simulations. Some of the residues formed more than one interaction with the ligand. This is represented by a darker shade of orange, according to the scale to the right of the plot. As shown in Figure 5d, ZINC1617939 displayed three distinct interactions with the Arg392 residue (hydrophobic, ionic, and water-bridge) as well as Asp507 (H-bond, ionic, and water-bridge) throughout the simulation time. Meanwhile, the interaction of the ligand with Glu510 was observed to be <50% of the simulation time.

**Figure 6:**
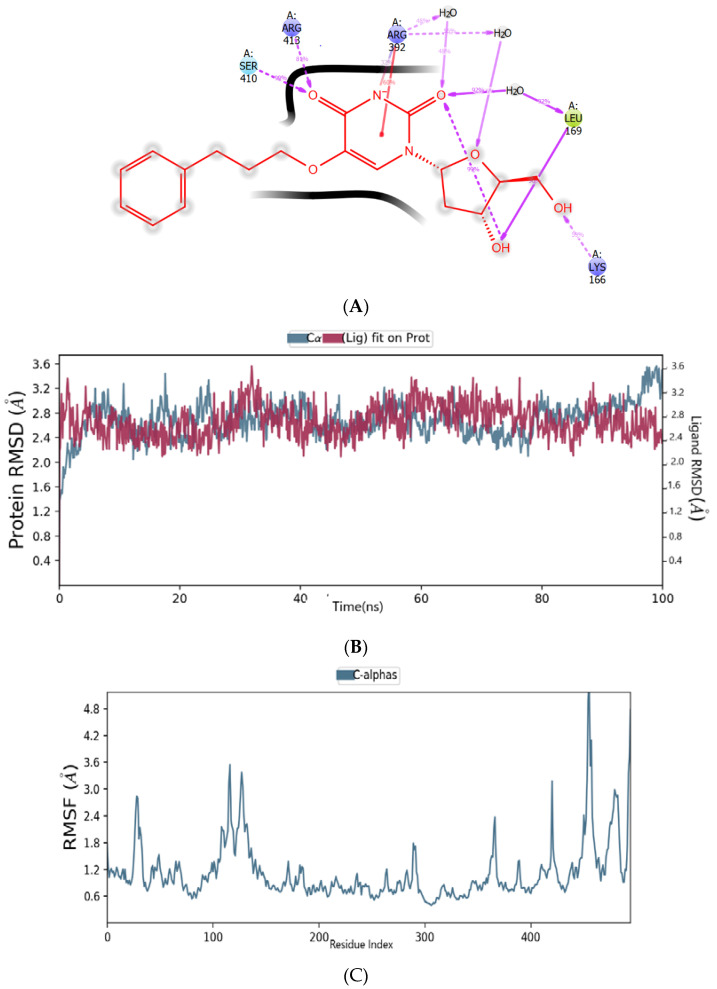

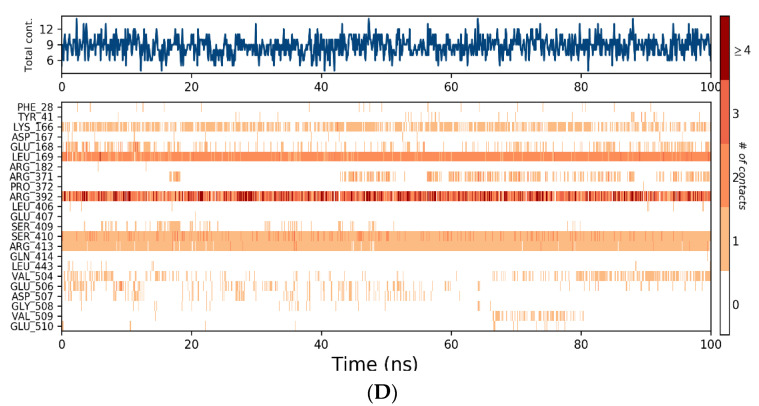
(**A**) The binding mode of ZINC1642549 in the active pocket of HNV-RdRp receptor after MD simulations; (**B**) The plot of root-mean-square deviation (RMSD); (**C**) The plot root-mean-square fluctuation (RMSF); (**D**) A timeline representation of the interactions and contacts of ZINC1642549 in the active site of HNV RdRp receptor.

In the binding mode of ZINC1642549 in the HNV RdRp receptor’s active pocket after the molecular simulation, we noticed that ZINC1642549 interacts with the residues, albeit with abridged proneness compared to the interaction before simulation. Besides, the 30% MD trajectory displayed the absence of a salt bridge and ionic interaction with Arg392, an exceptional attribute of the ZINC1617939 docked complex. The emerged results indicated the H-bond formation between the two-carbonyl oxygen atom of pyrimidinedione moiety and Leu169, Arg392, Arg413, and Ser410, as shown in Figure 6a. The negatively charged nitrogen atom H-bonded with Arg413, whereas the 3,4-hydroxyl of the nucleoside formed an H-bond interaction with residue Leu169 and Lys166, respectively. Interestingly, the negatively charged nitrogen atom on the pyrimidinedione moiety formed a salt bridge interaction with residue Arg413. Besides, salt bridge interactions with residue Arg413 and Arg392 have been reported to be critical interactions which mediate the binding [37]. High RMSD deviation was observed between 68 and 72 ns of the simulation time (Figure 6b), whereas the compound lacks interactions with residues with a high fluctuation. (Figure 6c). A timeline representation of the interactions and contacts (H-bonds, hydrophobic, ionic, water bridges) is shown in Figure 6d. The top panel shows the number of specific contacts the protein makes with the ligand throughout the trajectory. The bottom panel shows which residues interact with the ligands during the simulations. Some of the residues formed more than one interaction with the ligands. This is represented by a darker shade of orange, according to the scale to the right of the plot. As shown in Figure 6d, ZINC1642549 displayed three distinct interactions with the Arg392 residue (hydrophobic, ionic, H-bonds, and water-bridge) throughout the simulation time. In contrast, the hydrophobic contacts of Val509 and Phe28 with ZINC1642549 occur in a tiny fraction of time.

**Figure 7:**
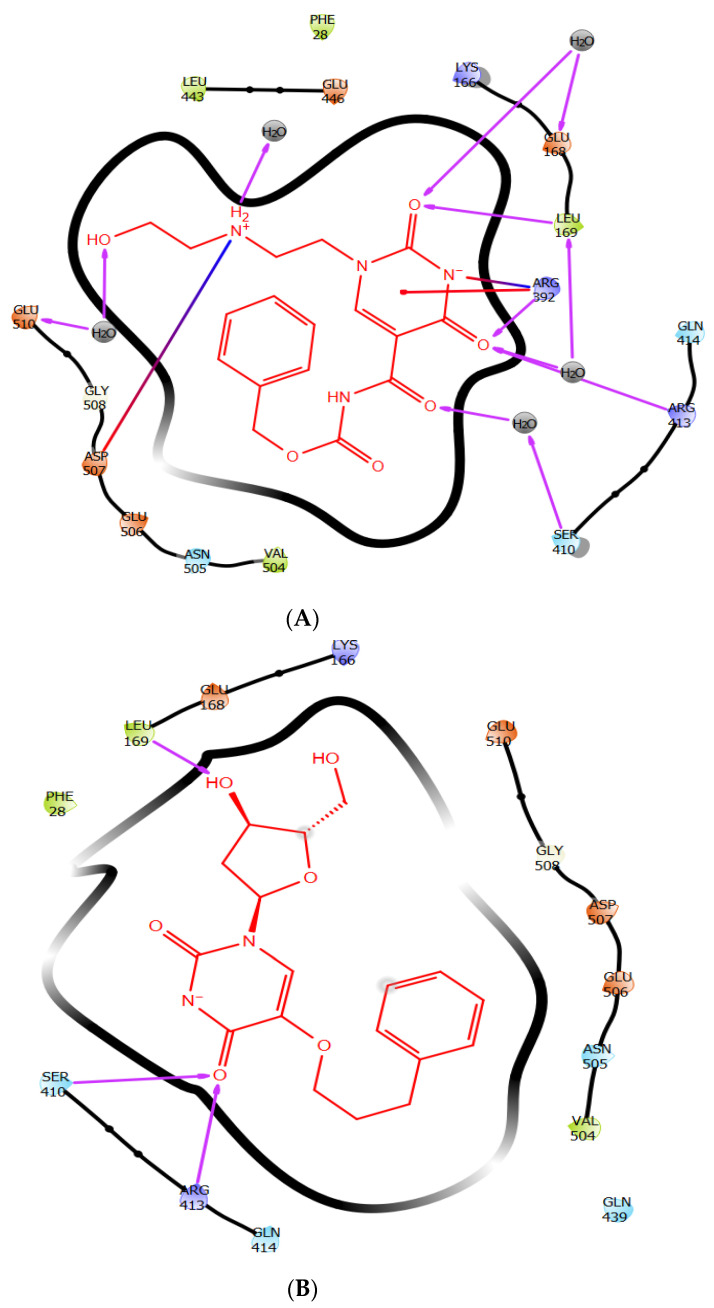
The representative structure of (**A**) ZINC1617939 and (**B**) ZINC1642549-protein complex from cluster 1, obtained from the MD trajectory.

The clustering of MD simulation frames was performed using the Desmond trajectory frame clustering to further establish and substantiate the MD simulation results in corroboration with the IFD results and rationalize the non-bonded interactions that stabilize the ligand within the HNV binding site. The frames used to calculate the RMSDs for the clustering are taken from the trajectory at a specified interval. The RMSD matrix is further used in the affinity propagation clustering method with a specified number of clusters. The results detailed the representative frame from each cluster. Based on the RMSD of backbone atoms, the binding site conformation was clustered into four different conformational groups, keeping frequency at 10. Figure 7 shown the representative protein–ligand complex from the first cluster for ZINC1617939 and ZINC1642549; meanwhile, the structures from the remaining clusters are shown in Figures S9 and S10. The clustering results show that the two ligands are stabilized by probable non-bonded interactions between the ligand and polar, hydrophobic, and charged amino acid side chains, as well as water within 4 Å of the ligands. ZINC1617939 is stabilized by various numbers of H-bond contacts with water molecules and amino acid side chains. Two salt bridges and one π-cation also stabilizes the complex. Meanwhile, ZINC1642549 has fewer H-bond interactions than ZINC1617939, and there is a lack of interactions with the water molecules. Notably, additional H-bond contacts were observed during the MD simulations compared to the IFD analysis.

For QM-MM calculations, the two-hit compounds and native ligands from the docked complexes were taken. The imperative orbitals in molecules for reactivity are the two frontier orbitals: the highest occupied molecular orbital (HOMO) and the lowest unoccupied molecular orbital (LUMO). The spatial distribution of HOMO suggests that it is the region of the ligand responsible for nucleophilic interaction during complex formation. In contrast, LUMO’s distribution denotes the region of ligand responsible for electrophilic interaction during complex formation. HOMO values are correlated to the ionization potential (IP), whereas the LUMO values are correlated to an electron affinity (EA). The disparity in HOMO and LUMO energy, known as HOMO-LUMO gap energy, implies the electronic excitation energy, which is crucial for predicting compounds’ molecular reactivity and stability. The results from QM-MM calculations are shown in the supporting document.

Among the hit compounds, the predicted RMSD of ZINC1617939 and ZINC1642549 were comparable to the CMX521 ligand. The interaction bar charting the representation of all the hit compounds and CMX521 are presented in the supporting document.

Interestingly, the two best lead compounds selected belong to the pyrimidinedione nucleoside analog, a prominent class with two nitrogen atoms and two substituted carbonyl groups in their six-membered ring. Pyrimidinedione has drawn significant interest in recent years. The structural rigidity and remarkable physicochemical properties have conferred their broad-spectrum activity and therapeutic potentials, thus maintaining many synthesized compounds’ targeted scaffolds. Few examples of the synthesized compound with pyrimidinedione moiety are therefore discussed. 5-Fluorouracil (5-FU) is an antimetabolite chemotherapeutic agent. It is used to treat various forms of cancers, such as colorectal, breast, head and neck, pancreas, and stomach cancers. The water solubility nature of this drug has made it administered intravenously, albeit that there is a necessity for its conversion to nucleotide level to exert its effect [38]. Trifluridine is an antiviral agent used to prevent and treat deeper eye infections, such as herpetic iritis and stromal keratitis caused by the herpes simplex virus types 1 and 2. Meanwhile, the combination therapy of this thymidine-based nucleoside analog with tipiracil potentiate trifluridine’s antitumor effectiveness and enables oral administration in the clinical setting [39]. Besides, 6-hydroxy-2-methylthiazolo [4,5-d]pyrimidine-5,7(4*H*,6*H*)- dione (NSC116565) has been identified as potential lead compound for anti-tuberculosis drug. NSC116565 hinders the growth of H37Ra and H37Rv strains of mycobacterium tuberculosis with MIC50 values of 2.93 µM and 6.06 µM, respectively [40]. Hence, we deduce that developing the potent and non-cytotoxic lead compounds ZINC1617939 and ZINC1642549 as a drug candidate for treating HNV infection will be effective. Apart from compounds ZINC6425208, ZINC5887658, and ZINC32068149, which displayed unbalanced backbone conformation with HNV RdRp protein during the simulation studies, compounds ZINC1617939 and ZINC1642549 can efficiently inhibit HNV RdRp. The study has shortcomings because of the lack of experimentation. Nevertheless, the results from this research provided a remarkable theoretical basis for the further chemical synthesis, structural characterization, and biological activity of the two compounds against HNV RdRp. We recommend that these compounds should be experimentally investigated for further validation.

## 4. Conclusions

Giving in to structure-based virtual screening, molecular docking, and molecular dynamics, compounds ZINC1617939 and ZINC1642549 can efficiently inhibit human norovirus. In contrast, ZINC6425208, ZINC5887658, and ZINC32068149 cannot be recommended because of instability during the trajectory period. The chemical structure variation of ZINC1617939 and ZINC1642549 for further optimization is a promising approach to developing an anti-norovirus agent. The ligand interaction results show that polar groups and amine with keen hydrogen bonding plausible will be vital for inhibitory potency. Probing these observable facts will allow structure-activity-guided molecular modification to afford more effective anti-norovirus agents to improve human health without a doubt.

## Figures and Tables

**Table 1:** Pharmacokinetic properties of the lead compounds using Qikprop.

Entry	Compound ID	QPlogPw	QPlogPo/w	QPlogS	QPlogHERG	QPPCaco	QPlogBB	QPlogKp	QPlogKhsa	HOR
1	ZINC1617939	12.889	1.000	−2.603	−5.463	6.582	−2.253	−7.077	−0.638	2
2	ZINC6425208	8.949	2.812	−4.174	−4.815	246.337	−1.278	−3.233	−0.127	3
3	ZINC1642549	15.413	1.320	−2.842	−4.503	223.103	−1.449	−5.618	−0.368	3
4	ZINC5887658	9.948	2.015	−4.306	−4.306	59.428	−0.308	−3.897	−0.369	3
5	ZINC32068149	19.353	2.339	−4.137	−5.539	144.709	−0.420	−4.708	−0.227	3

QPlogPw: water/gas partition (4.0–45.0); logPo/w: predicted octanol/water partition coefficient (−2.0–6.5); log*S*: Predicted aqueous solubility, logS. S in mol dm^−3^ is the concentration of the solute in a saturated solution that is in equilibrium with the crystalline solid (−6.5–0.5); log *HERG*: the predicted IC_50_ value for the blockage of HERG K^+^ channels (concern below −5); Caco-2: cell membrane permeability (<25 poor >500 good); log BB: logarithm of predicted blood/brain barrier partition coefficient (−3.0–1.2); log *K*_p_: predicted skin permeability and 95% of drugs: (−8–1); log BB: logarithm of predicted blood/brain barrier partition coefficient (−3.0–1.2); log *K_HSA_*: logarithm of predicted binding constant to human serum albumin (−1.5–1.5); Human Oral Absorption (HOR)- 1-low, 2-medium, 3-high.

**Table 2:** The IFD results of the potent compounds that passed through ADMET.

Entry	Compound ID	Glide Score (kcal/mol)	Glide Emodel (kcal/mol)	Glide Energy (kcal/mol)	IFD Score (kcal/mol)
1	ZINC1617939	−13.027	−81.298	−60.302	−1045.910
2	ZINC6425208	−9.226	−52.870	−38.120	−1040.540
3	ZINC1642549	−9.003	−81.682	−54.214	−1040.180
4	ZINC5887658	−9.744	−50.500	−45.429	−1042.37
5	ZINC32068149	−10.507	−67.805	−50.600	−1042.18
4	CMX521	−7.886	−79.970	−57.383	−1041.660

## Data Availability

The data presented in this study are available in supplementary material.

## Supplementary Materials

The following are available online, Figure S1. The active site of HNV-RdRp with the surface of (A) acceptor, (B) donor, (C) hydrophilic, (D) hydrophobic in the presence of co-crystallized ligand (PPNDS). Figure S2 The 3-Dimensional representation compound PPND and ZINC1617939 in the binding pocket of HNV RdRp Figure S3. The 3-Dimensional representation compound ZINC6425208 in the binding pocket of HNV RdRp Figure S4 The 3-Dimensional representation compound ZINC1642549 in the binding pocket of HNV RdRp. Figure S5. Frontier molecular orbitals plots (A) HOMO and (B) LUMO of the hit compounds ZINC1617939 obtained from the SPE calculation. Figure S6. Frontier molecular orbitals plots (A) HOMO and (B) LUMO of the hit compound ZINC1642549 obtained from the SPE calculation. Figure S7. Molecular Electrostatic Potential surface plots of the hit compounds, ZINC1617939, and ZINC1642549, were obtained from the SPE calculation. Figure S8. Interaction fractions plot of the hit compounds, CMX521, ZINC1617939, and ZINC1642549, after MD. Figure S9. The representative structure of ZINC1617939 -protein complex from cluster 2-4, obtained from the MD trajectory. Figure S9. The representative structure of ZINC1642549 -protein complex from cluster 2–4, obtained from the MD trajectory

## Sample Availability

Samples of the compounds are not available from the authors.

## References

[1] Chhabra P., de Graaf M., Parra G.I., Chan M.C.-W., Green K., Martella V., Wang Q., White P.A., Katayama K., Vennema H. (2019). Updated classification of norovirus genogroups and genotypes. J. Gen. Virol..

[2] Ebenezer O., Jordaan M.A., Damoyi N., Shapi M. (2021). Discovery of Potential Inhibitors for RNA-Dependent RNA Polymerase of Norovirus: Virtual Screening, and Molecular Dynamics. Int. J. Mol. Sci..

[3] Mastrangelo E., Pezzullo M., Tarantino D., Petazzi R., Germani F., Kramer D., Robel I., Rohayem J., Bolognesi M., Milani M. (2012). Structure-based inhibition of Norovirus RNA-dependent RNA polymerases. J. Mol. Biol..

[4] Green K., Knipe D., Howley P. (2007). Caliciviridae: The noroviruses. Fields virology. Lippincott.

[5] Bányai K., Estes M.K., Martella V., Parashar U.D. (2018). Viral gastroenteritis. Lancet.

[6] Alsved M., Fraenkel C.-J., Bohgard M., Widell A., Söderlund-Strand A., Lanbeck P., Holmdahl T., Isaxon C., Gudmundsson A., Medstrand P. (2020). Sources of airborne norovirus in hospital outbreaks. Clin. Infect. Dis..

[7] Nenonen N.P., Hannoun C., Svensson L., Torén K., Andersson L.-M., Westin J., Bergström T. (2014). Norovirus GII. 4 detection in environmental samples from patient rooms during nosocomial outbreaks. J. Clin. Microbiol..

[8] Cortes-Penfield N.W., Ramani S., Estes M.K., Atmar R.L. (2017). Prospects and challenges in the development of a norovirus vaccine. Clin. Ther..

[9] Chang K.-O., George D.W. (2007). Interferons and ribavirin effectively inhibit Norwalk virus replication in replicon-bearing cells. J. Virol..

[10] Rocha-Pereira J., Jochmans D., Dallmeier K., Leyssen P., Cunha R., Costa I., Nascimento M., Neyts J. (2012). Inhibition of norovirus replication by the nucleoside analogue 2′-C-methylcytidine. Biochem. Biophys. Res. Commun..

[11] Kolawole A.O., Rocha-Pereira J., Elftman M.D., Neyts J., Wobus C.E. (2016). Inhibition of human norovirus by a viral polymerase inhibitor in the B cell culture system and in the mouse model. Antivir. Res..

[12] Furuta Y., Takahashi K., Fukuda Y., Kuno M., Kamiyama T., Kozaki K., Nomura N., Egawa H., Minami S., Watanabe Y. (2002). In vitro and in vivo activities of anti-influenza virus compound T-705. Antimicrob. Agents Chemother..

[13] Barnard D.L., Day C.W., Bailey K., Heiner M., Montgomery R., Lauridsen L., Chan P.K., Sidwell R.W. (2006). Evaluation of immunomodulators, interferons and known in vitro SARS-coV inhibitors for inhibition of SARS-coV replication in BALB/c mice. Antivir. Chem. Chemother..

[14] Gowen B.B., Smee D.F., Wong M.-H., Hall J.O., Jung K.-H., Bailey K.W., Stevens J.R., Furuta Y., Morrey J.D. (2008). Treatment of late stage disease in a model of arenaviral hemorrhagic fever: T-705 efficacy and reduced toxicity suggests an alternative to ribavirin. PLoS ONE.

[15] Rocha-Pereira J., Jochmans D., Dallmeier K., Leyssen P., Nascimento M., Neyts J. (2012). Favipiravir (T-705) inhibits in vitro norovirus replication. Biochem. Biophys. Res. Commun..

[16] Arias A., Thorne L., Goodfellow I. (2014). Favipiravir elicits antiviral mutagenesis during virus replication in vivo. Elife.

[17] Ferla S., Netzler N.E., Ferla S., Veronese S., Tuipulotu D.E., Guccione S., Brancale A., White P.A., Bassetto M. (2018). In silico screening for human norovirus antivirals reveals a novel non-nucleoside inhibitor of the viral polymerase. Sci. Rep..

[18] Harmalkar D.S., Lee S.-J., Lu Q., Kim M.I., Park J., Lee H., Park M., Lee A., Lee C., Lee K. (2019). Identification of novel non-nucleoside vinyl-stilbene analogs as potent norovirus replication inhibitors with a potential host-targeting mechanism. Eur. J. Med. Chem..

[19] Croci R., Pezzullo M., Tarantino D., Milani M., Tsay S.-C., Sureshbabu R., Tsai Y.-J., Mastrangelo E., Rohayem J., Bolognesi M. (2014). Structural bases of norovirus RNA dependent RNA polymerase inhibition by novel suramin-related compounds. PLoS ONE.

[20] https://wiki.nci.nih.gov/display/ncidtpdata/aids+antiviral+screen+data.

[21] (2020). LigPrep, S..

[22] Greenwood J.R., Calkins D., Sullivan A.P., Shelley J.C. (2010). Towards the comprehensive, rapid, and accurate prediction of the favorable tautomeric states of drug-like molecules in aqueous solution. J. Comput. -Aided Mol. Des..

[23] Shelley J.C., Cholleti A., Frye L.L., Greenwood J.R., Timlin M.R., Uchimaya M. (2007). Epik: A software program for pK a prediction and protonation state generation for drug-like molecules. J. Comput. -Aided Mol. Des..

[24] Kokic G., Hillen H.S., Tegunov D., Dienemann C., Seitz F., Schmitzova J., Farnung L., Siewert A., Höbartner C., Cramer P. (2021). Mechanism of SARS-CoV-2 polymerase stalling by remdesivir. Nat. Commun..

[25] Friesner R.A., Banks J.L., Murphy R.B., Halgren T.A., Klicic J.J., Mainz D.T., Repasky M.P., Knoll E.H., Shelley M., Perry J.K. (2004). Glide: A new approach for rapid, accurate docking and scoring. 1. Method and assessment of docking accuracy. J. Med. Chem..

[26] Dixon S.L., Smondyrev A.M., Knoll E.H., Rao S.N., Shaw D.E., Friesner R.A. (2006). PHASE: A new engine for pharmacophore perception, 3D QSAR model development, and 3D database screening: 1. Methodology and preliminary results. J. Comput. -Aided Mol. Des..

[27] Kuhn B., Kollman P.A. (2000). Binding of a diverse set of ligands to avidin and streptavidin: An accurate quantitative prediction of their relative affinities by a combination of molecular mechanics and continuum solvent models. J. Med. Chem..

[28] (2020). Inducedfit Docking, S..

[29] Murphy R.B., Philipp D.M., Friesner R.A. (2000). A mixed quantum mechanics/molecular mechanics (QM/MM) method for large-scale modeling of chemistry in protein environments. J. Comput. Chem..

[30] Philipp D.M., Friesner R.A. (1999). Mixed ab initio QM/MM modeling using frozen orbitals and tests with alanine dipeptide and tetrapeptide. J. Comput. Chem..

[31] (2020). Jaguar, S..

[32] Jorgensen W.L., Chandrasekhar J., Madura J.D., Impey R.W., Klein M.L. (1983). Comparison of simple potential functions for simulating liquid water. J. Chem. Phys..

[33] Ali A., Vijayan R. (2020). Dynamics of the ACE2–SARS-CoV-2/SARS-CoV spike protein interface reveal unique mechanisms. Sci. Rep..

[34] Halgren T.A. (2009). Identifying and characterizing binding sites and assessing druggability. J. Chem. Inf. Modeling.

[35] Lipinski C.A. (2004). Lead-and drug-like compounds: The rule-of-five revolution. Drug Discov. Today: Technol..

[36] (2020). QikProp, S..

[37] Tarantino D., Pezzullo M., Mastrangelo E., Croci R., Rohayem J., Robel I., Bolognesi M., Milani M. (2014). Naphthalene-sulfonate inhibitors of human norovirus RNA-dependent RNA-polymerase. Antivir. Res..

[38] Pinedo H.M., Peters G. (1988). Fluorouracil: Biochemistry and pharmacology. J. Clin. Oncol..

[39] Kang C., Dhillon S., Deeks E.D. (2019). Trifluridine/tipiracil: A review in metastatic gastric cancer. Drugs.

[40] Lin X., Kurz J., Patel K., Wun S.J., Hussein W., Lonhienne T., West N.P., McGeary R.P., Schenk G., Guddat L.W. (2020). Discovery of a pyrimidine-dione derivative with potent inhibitory activity against Mycobacterium tuberculosis ketol-acid reductoisomerase. Chemistry–A Eur. J..

