# Supplementary material for: Unveiling of Pyrimidindinones as Potential Anti-Norovirus Agents—A Pharmacoinformatic-Based Approach
Source: Molecules. 2022 Jan 7;27(2):380. doi: 10.3390/molecules27020380 (PMC8777711; doi:10.3390/molecules27020380)
Supplement: Supplementary file 1 [file molecules-27-00380-s001.zip › molecules-1488219-supplementary.pdf]

Pyrimidindinones as potential anti-norovirus agents - Pharmacoinformatic-based approach

Oluwakemi Ebenezer*, Maryam A Jordaan, Nkululeko Damoyi and Michael Shapi
Faculty of Natural Science, Department of Chemistry, Mangosuthu University of Technology, 511 Mangosuthu Highway, Durban, 4000, South Africa
*Correspondence: re.korede@gmail.com

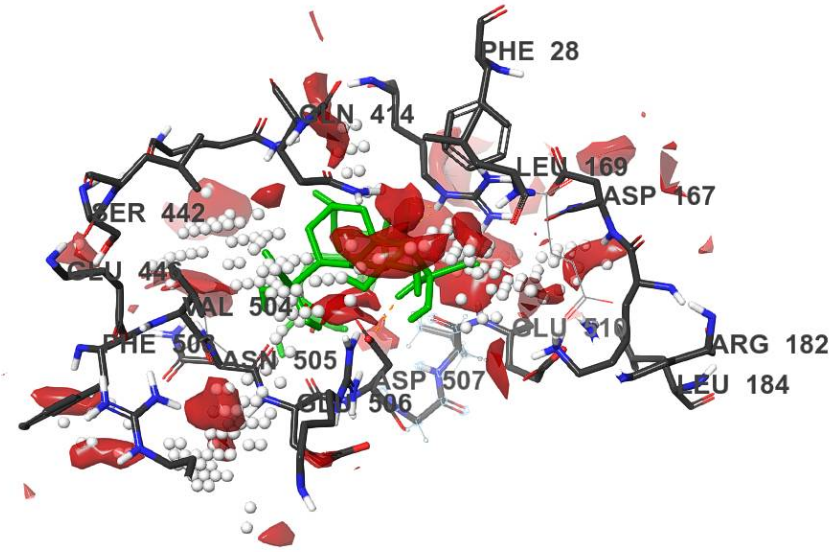

A

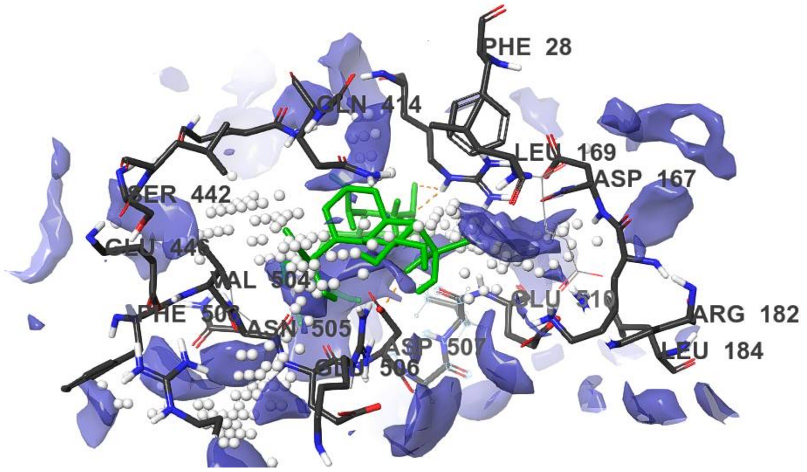

B

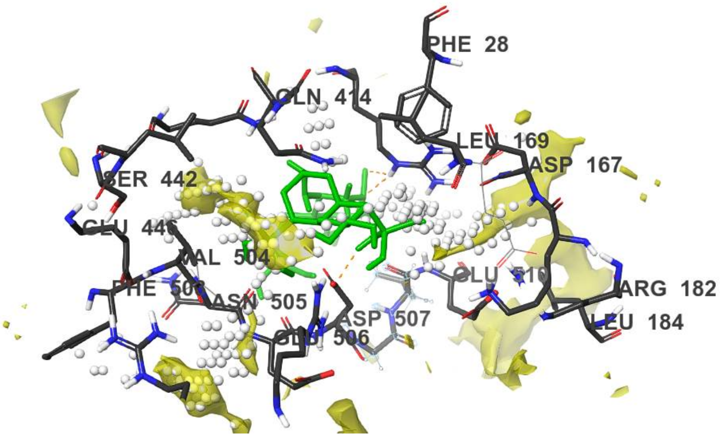

C

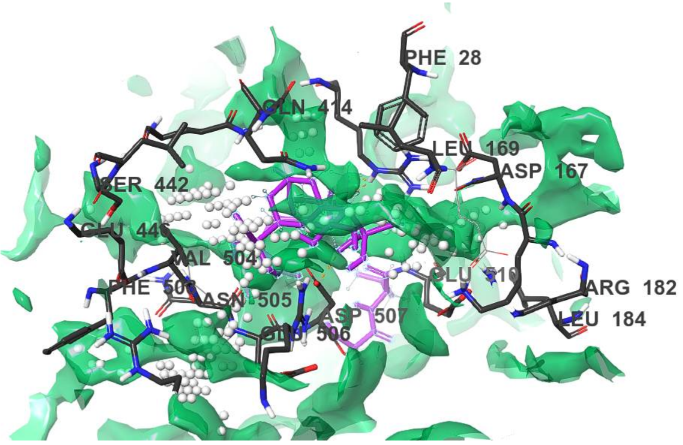

D

Figure S1. The active site of HNV-RdRp with the surface of (A) acceptor, (B) donor, (C) hydrophilic, (D) hydrophobic in the presence of co-crystallized ligand (PPNDS).

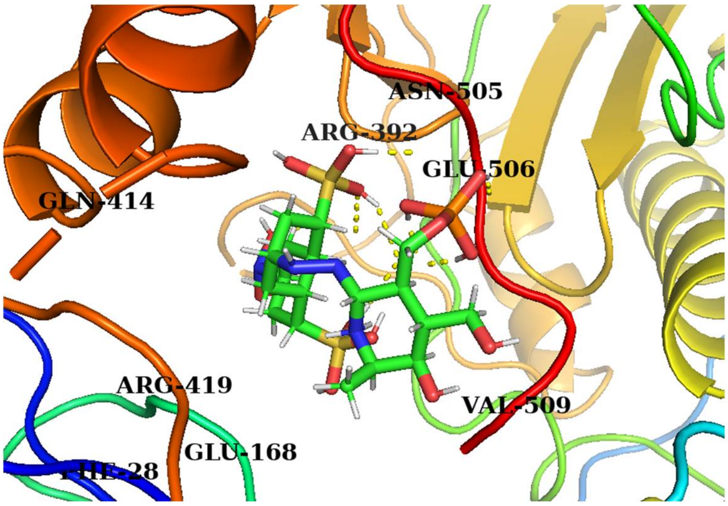

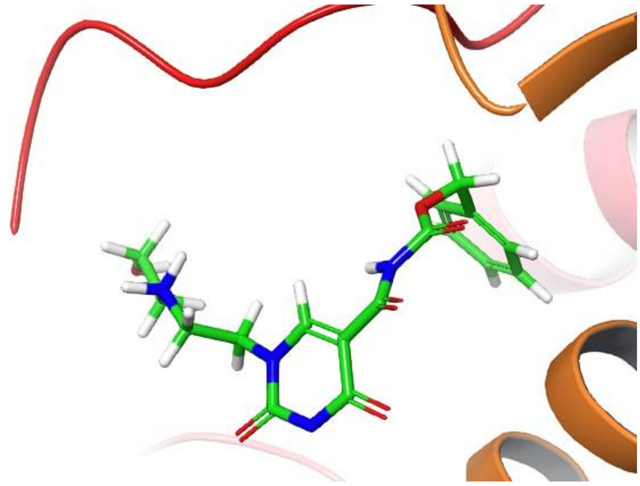

Figure S2 The 3-Dimensional representation compound PPND and ZINC1617939 in the binding pocket of HNV RdRp

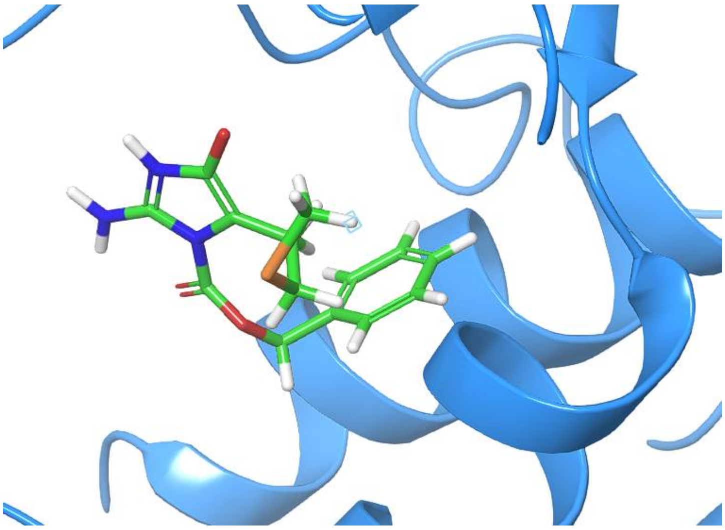

Figure S3. The 3-Dimensional representation compound ZINC6425208 in the binding pocket of HNV RdRp

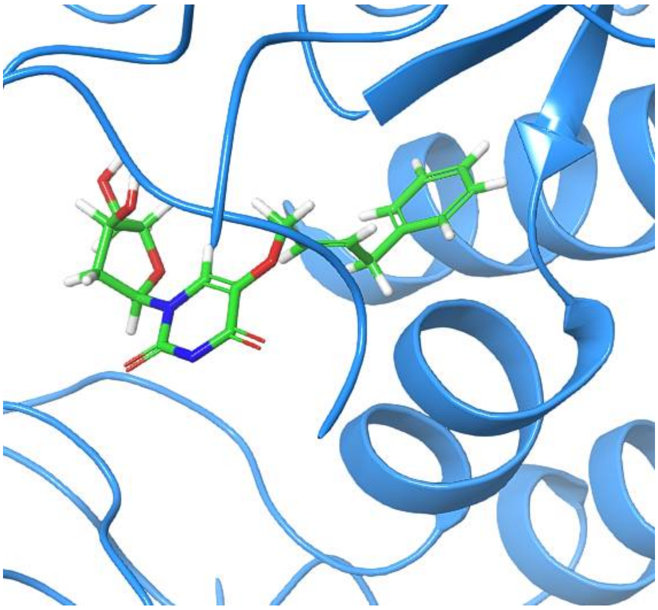

A

Figure S4 The 3-Dimensional representation compound ZINC1642549 in the binding pocket of HNV RdRp.

HOMO and LUMO plots are shown in Figure S5 and S6 for the hit compounds. The positive electron densities are mapped in red, while the negative electron densities are mapped in blue. The energy gap (Egap) of the hit compounds correspond to 4.731 eV (ZINC1617939, HOMO = –5.278 eV and LUMO = –0.547eV), 2.392eV (ZINC6425208, HOMO = –3.737eV and LUMO = –1.345 eV) and 4.520 eV (ZINC1642549, HOMO = –3.276 eV and LUMO = 1.244 eV). Besides, compound ZINC6425208 exhibited the lowest energy gap, and the highest energy gap was observed in compound ZINC1617939. Meanwhile, a small energy gap suggests less stability as well as high reactivity of the ligand. Interestingly, the hits' energy gap values were lesser than the co-crystal ligand, denoting the lesser stability and high chemical reactivity of PPNDS (HOMO = –7.268 eV and LUMO = –6.451 eV, Egap = 0.817 eV). Furthermore, the electrostatic potential surface scale increases in order of blue > green > yellow > orange > red. The depicted blue color implies the strongest repulsion, red indicates attraction, and green indicates a neutral electrostatic potential. The red color denotes the most negative electrostatic potential region. Blue denotes the most positive electrostatic potential region. The green color denotes the region of zero potential, as shown in Figure 7. The hit compounds' molecular electrostatic potential (MEP) surface revealed negative potentials on the carbonyl functional group. The electron density of the carbonyl group's oxygen atom enhances it to act out as an H-bond acceptor, which helps the compounds sustain better attraction toward the HNV RdRp receptor's active site thus can also contribute to an increase in their inhibitory activity. The green color explained the neutral electrostatic potential surface for the compounds. The compounds' MEP surface is accountable for their binding in the active sites of HNV RdRp, which also have negative electrostatic potential zones.

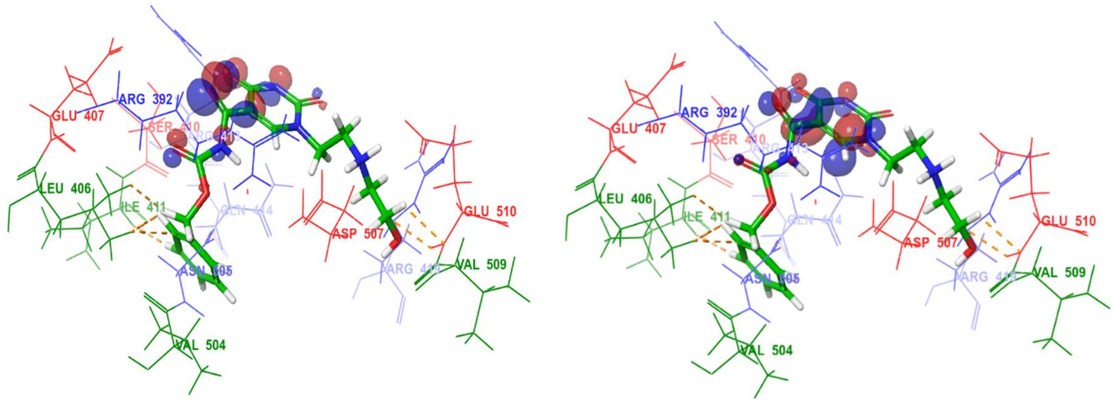

A B

Figure S5. Frontier molecular orbitals plots (A) HOMO and (B) LUMO of the hit compounds ZINC1617939 obtained from the SPE calculation.

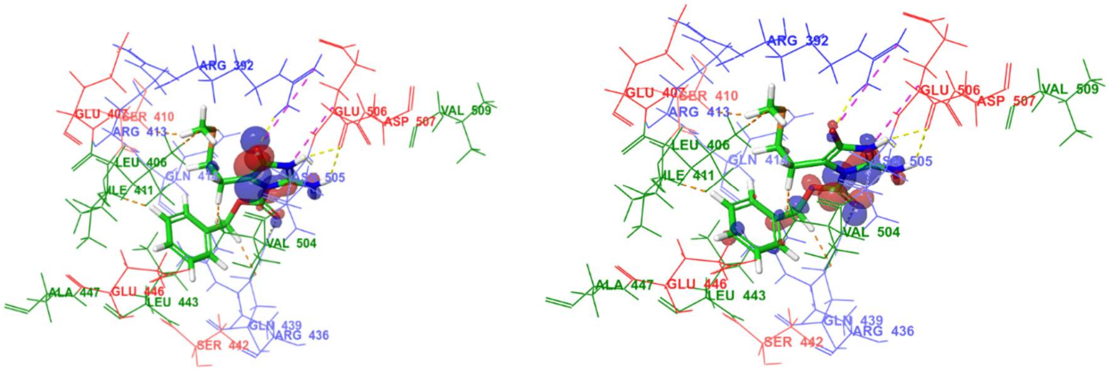

A B

ZINC6425208

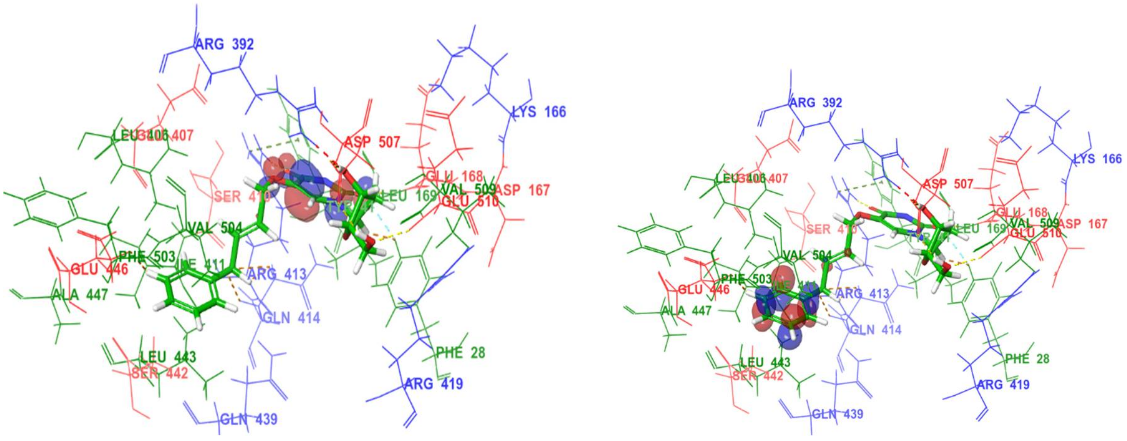

A B

ZINC1642549

Figure S6. Frontier molecular orbitals plots (A) HOMO and (B) LUMO of the hit compounds ZINC6425208, and ZINC1642549 obtained from the SPE calculation.

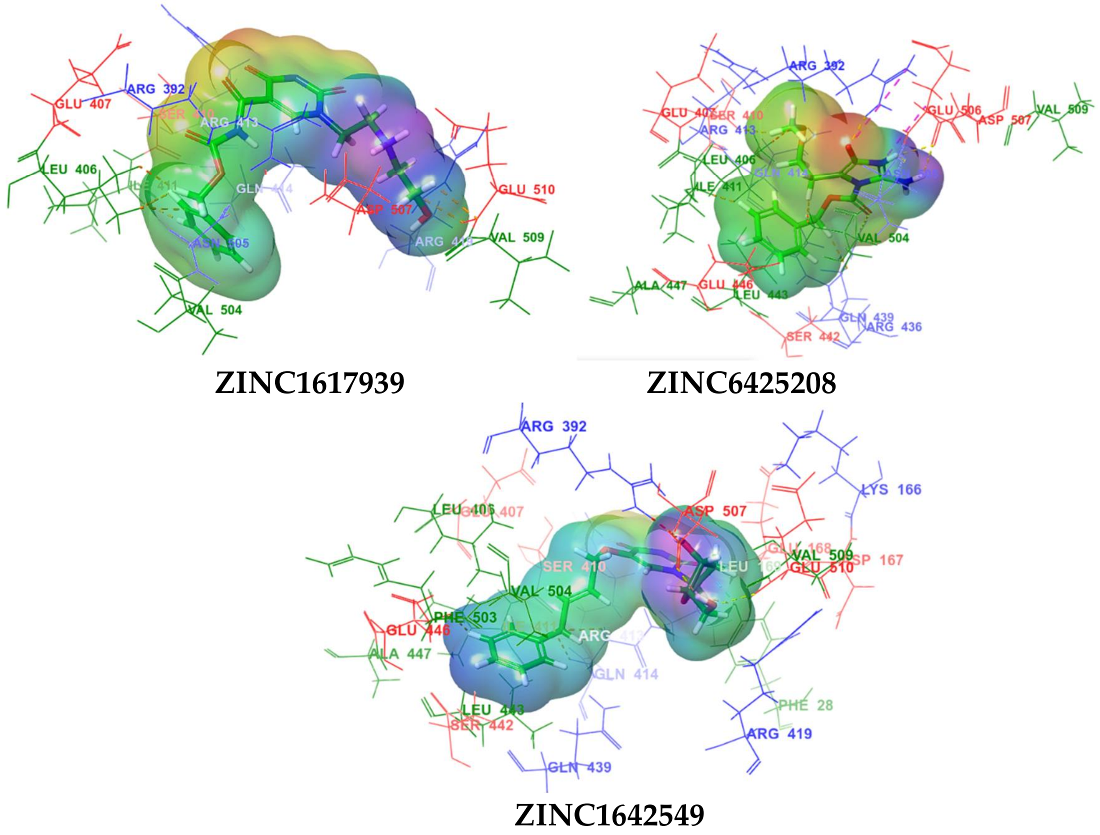

Figure S7. Molecular Electrostatic Potential surface plots of the hit compounds, ZINC1617939, ZINC6425208, and ZINC1642549, were obtained from the SPE calculation.

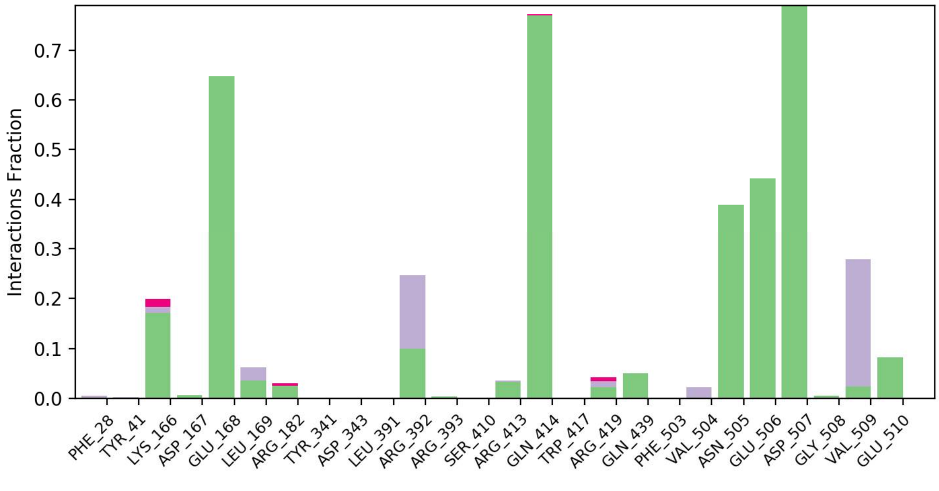

A

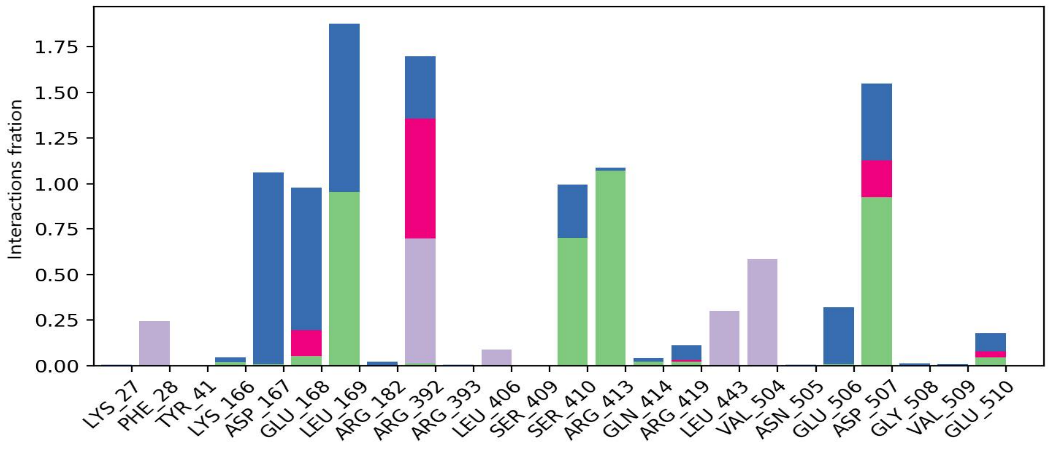

B

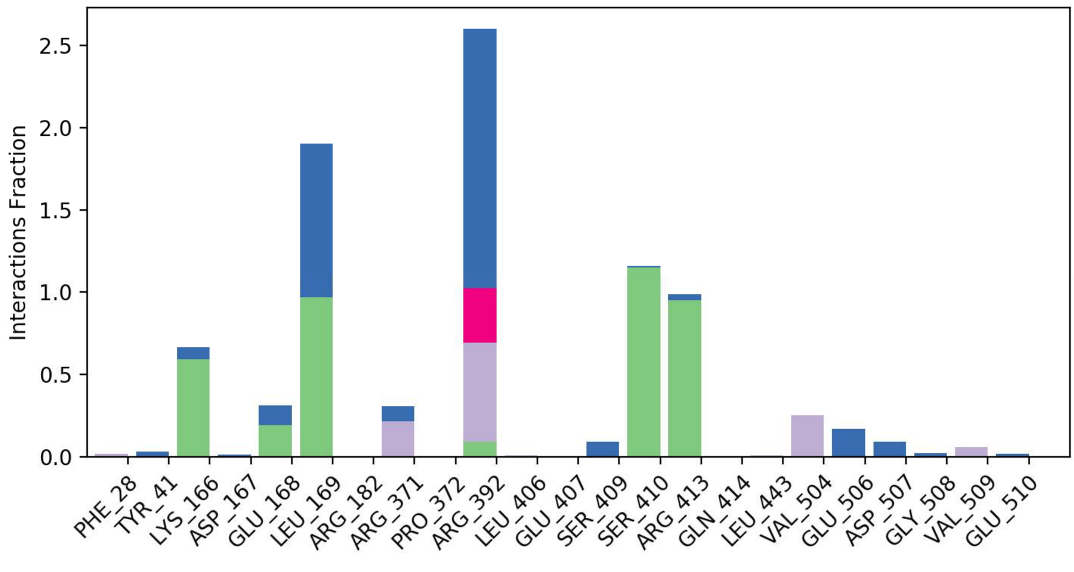

C

Figure S8. Interaction fractions plot of the hit compounds, CMX521, ZINC1617939, and ZINC1642549, after MD.

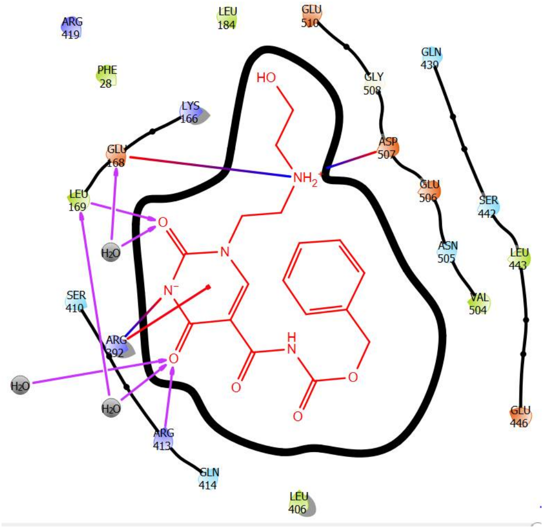

A

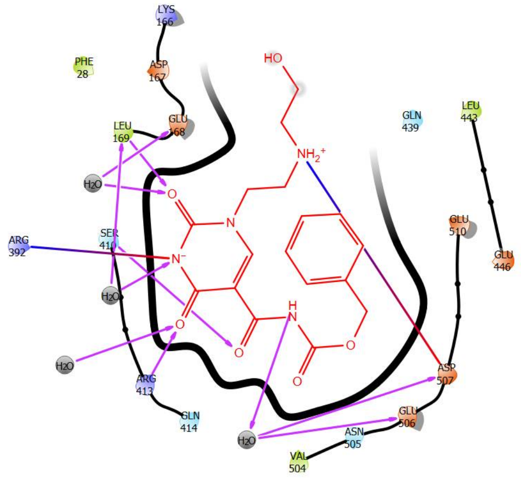

B

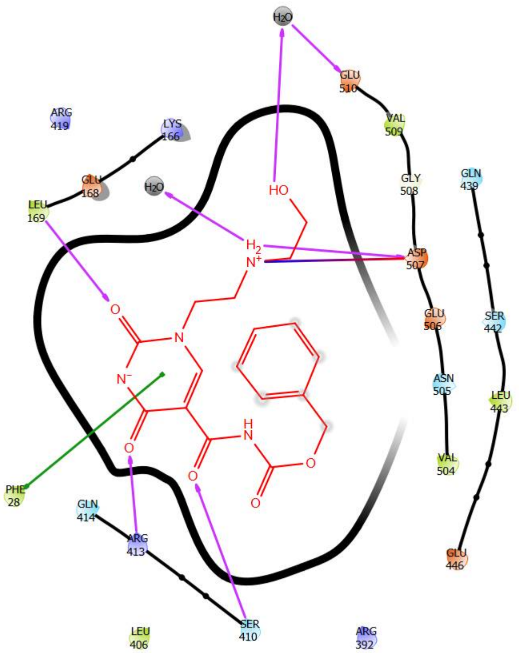

C

Figure S9. The representative structure of ZINC1617939 -protein complex from cluster 2-4, obtained from the MD trajectory.

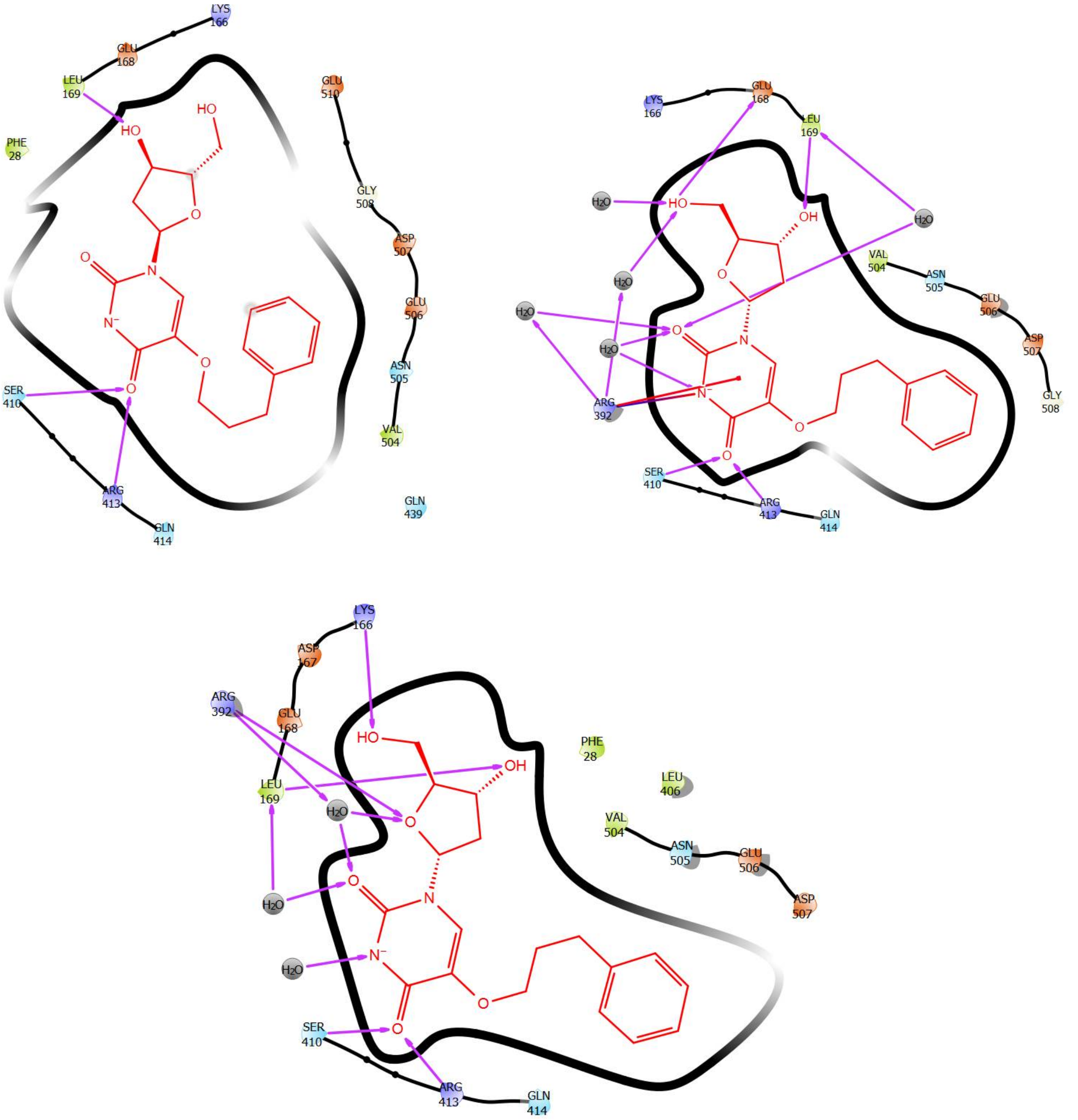

Figure S9. The representative structure of ZINC1642549 -protein complex from cluster 2-4, obtained from the MD trajectory
